# Supplementary material for: CREB Is Activated by the SCF/KIT Axis in a Partially ERK-Dependent Manner and Orchestrates Survival and the Induction of Immediate Early Genes in Human Skin Mast Cells
Source: Int J Mol Sci. 2023 Feb 18;24(4):4135. doi: 10.3390/ijms24044135 (PMC9966046; doi:10.3390/ijms24044135)
Supplement: Supplementary file 1 [file ijms-24-04135-s001.zip › ijms-2167468-supplementary.pdf]

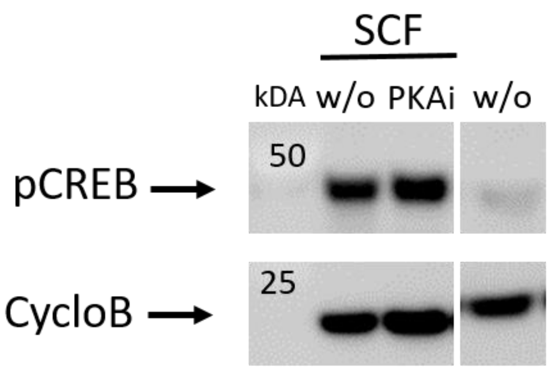

Figure S1: SCF elicited CREB phosphorylation occurs in a PKA-independent manner. Skin-derived MCs were pretreated with a PKA inhibitor (PKAi), then stimulated with SCF for 15 min; phosphorylation of CREB was detected by immunoblot. CycloB = Cyclophilin B (loading control). One of four independent experiments with comparable outcomes is shown.

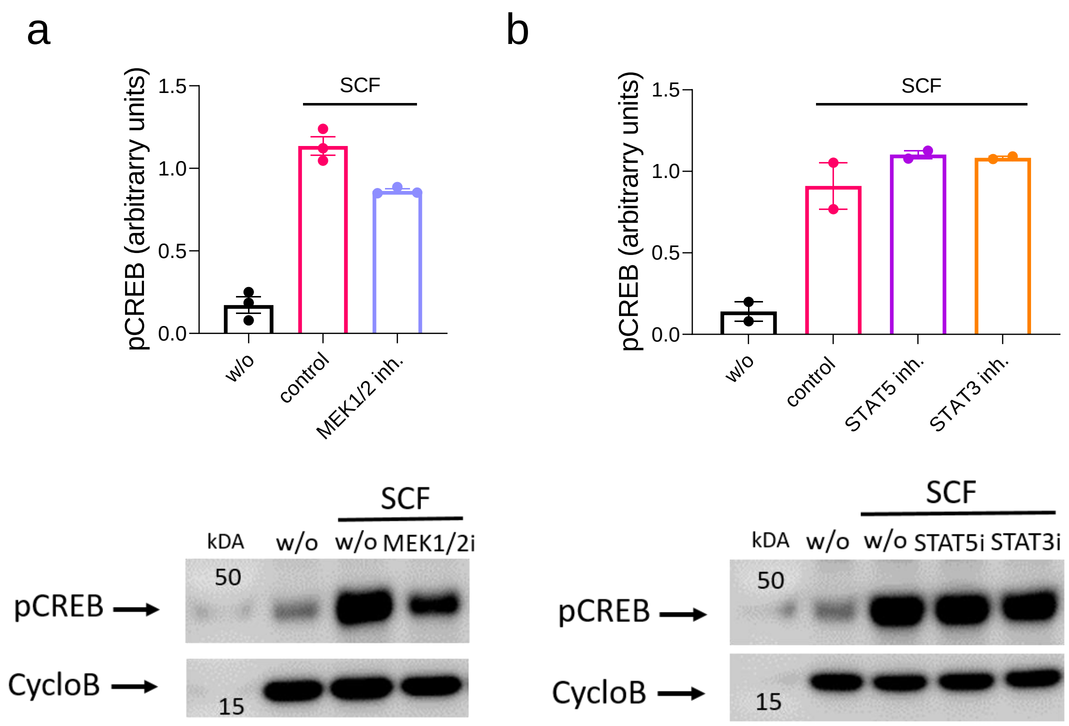

Figure S2: SCF-elicited CREB phosphorylation depends on MEK1/2 activity. (a, b) Skin-derived MCs were pretreated with different inhibitors, then stimulated with SCF for 15 min; phosphorylation of CREB was detected by immunoblot in whole-cell lysates. Upper panel: Image J based semi-quantification of the detected signal for pCREB normalized to the housekeeping protein Cyclophilin B (CycloB). Lower panel: Representative blots. (a) MEK1/2 inhibitor. (b) Inhibitors of STAT5 and STAT3 function.

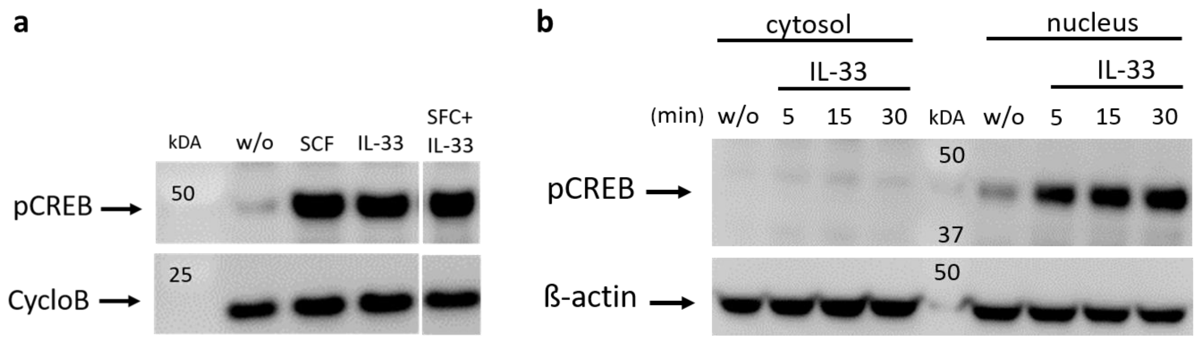

Figure S3: CREB incurs phosphorylation following IL-33-mediated stimulation. (a) MCs were treated with IL-33, SCF or IL-33+SCF for 15 min, and pCREB was detected by immunoblot as in main Figure 1. CycloB = cyclophilin B (loading control). One of two experiments with comparable outcomes is shown. (b) MCs were treated with IL-33 for the times given, cytosolic and nuclear fractions prepared separately, and pCREB detected by immunoblot as in main Figure 3. β-actin served as the loading control.

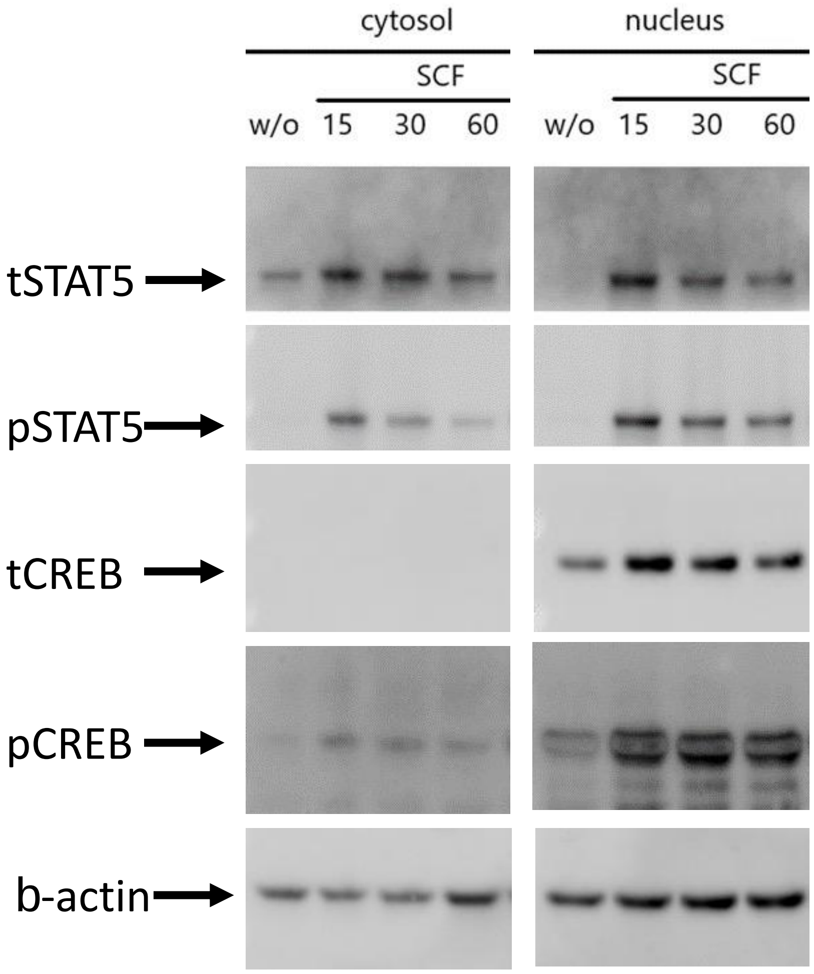

Figure S4: STAT5 is only cytoplasmic prior to stimulation, while CREB is constitutively nuclear. MCs were treated with SCF for the indicated times, and cytosolic and nuclear fractions prepared separately. Total (t) and phosphorylated (p) STAT5 and CREB were detected by immunoblot in both compartments. β-actin served as the loading control. Please note that the anti-tSTAT5 Ab binds more efficiently when STAT5 is also phosphorylated; this is the reason why there is seemingly more tSTAT5 in the cytoplasm after 15 and 30 min of SCF treatment, though there is substantial translocation to the nucleus at these times. The degree of phosphorylation on CREB also likely influences the binding of anti-total CREB antibody at least to some degree. One representative out of two blots with comparable outcome is shown.

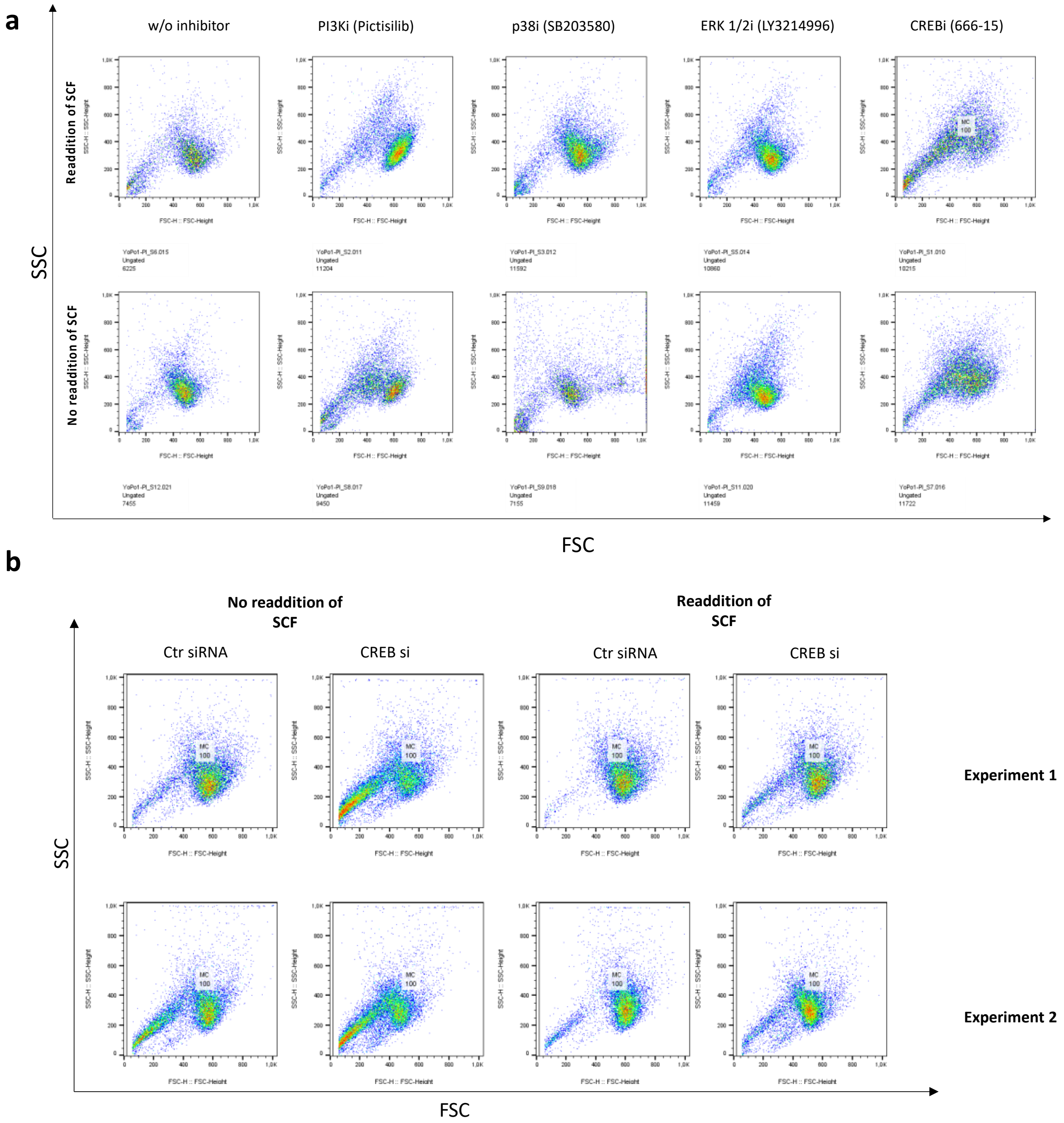

Figure S5: Scatterplots showing cell distribution pertaining to Main Figures (a) 5 and (b) 6. The cells were not gated, but instead 100% of the MC population were used to determine the proportion of apoptotic and viable cells. Note that the scatterplots nicely correspond with the YoPro/PI staining of the main Figures. SSC – side scatter, FSC – forward scatter, w/o – without.

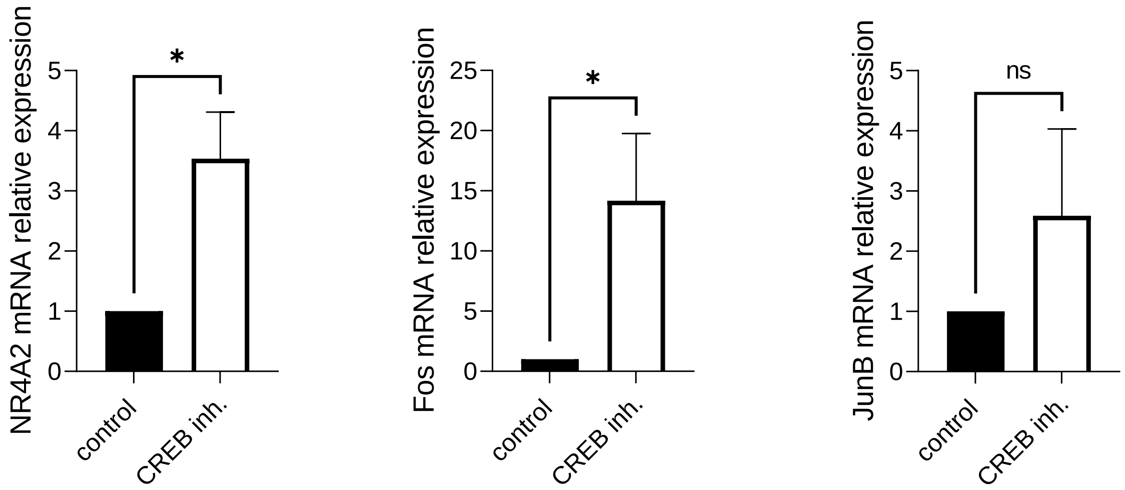

Figure S6: CREB inhibition slightly promotes low-level baseline expression of immediate-early genes. Skin-derived MCs were treated with the CREB inhibitor 666-15 for a total of 40 min; RT-qPCR was used to quantitate gene expression (normalized to several housekeeping genes). Control expression was set to 1, and the expression in the treated group is expressed as fold change. Mean ± SEM of 8 experiments (separate cultures) * $p<0.05$.

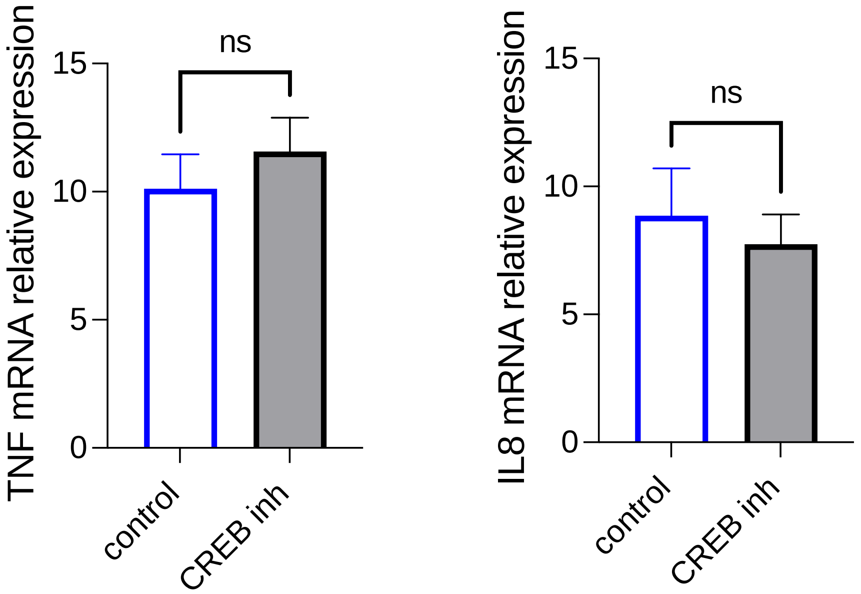

Figure S7: CREB does not contribute to SCF-triggered induction of *TNF* and *IL8*. Skin-derived MCs were pretreated with(out) the CREB inhibitor 666-15, then stimulated (or not) with SCF for 25 min; RT-qPCR was used to quantitate gene expression (normalized to several housekeeping genes). The fold-induction by SCF over control is depicted, exactly as in main Figure 7. Mean ± SEM of 4 experiments (separate cultures). ns – not significant.
